# Association study of *HIF-1α* rs11549465 and *VEGF* rs3025039 genetic variants with diabetic retinopathy in Egyptian patients: crosslinks with angiogenic, inflammatory, and anti-inflammatory markers

**DOI:** 10.1186/s43141-022-00401-9

**Published:** 2022-08-15

**Authors:** Mahmoud K. Mohamed, Azza A. Atef, Leqaa A. Moemen, Amira A. Abdel Azeem, Islam A. Mohalhal, Alshaimaa M. Taha

**Affiliations:** 1 Biochemistry Unit, Research Institute of Ophthalmology, Giza, Egypt; 2 Department of Biochemistry, Faculty of Science, Ain Shams University, Cairo, Egypt; 3 Genetic ophthalmology, Research Institute of Ophthalmology, Giza, Egypt; 4 Surgical Retina, Research Institute of Ophthalmology, Giza, Egypt

**Keywords:** T2DM, AGEP, VCAM-1, VEGF, CTRP3, NPDR, PDR, Risk factor

## Abstract

**Background:**

Genetic factors are implicated in the progression of DR—a global cause of blindness. Hence, the current work investigated the association of *HIF-1α* rs11549465 and *VEGF* rs3025039 genetic variants with the different stages of retinopathy among T2DM Egyptian patients. The crosslinks of these variants were explored with angiogenesis (VEGF), inflammation (AGEP and VCAM-1), and anti-inflammation (CTRP3) markers. Two hundred eighty-eight subjects were recruited in this study: 72 served as controls and 216 were having T2DM and were divided into diabetics without retinopathy (DWR), diabetics with non-proliferative retinopathy (NPDR), and diabetics with proliferative retinopathy (PDR). The genetic variants were analyzed using PCR-RFLP and their associations with NPDR and PDR were statistically tested. The circulating levels of AGEP, VCAM-1, HIF-1α, VEGF, and CTRP3 were assayed followed by analyzing their associations statistically with the studied variants.

**Results:**

Only *HIF-1α* rs11549465 genetic variant (recessive model) was significantly associated with the development of NPDR among T2DM patients (*p* < 0.025) with a significant correlation with the circulating HIF-1α level (*p* < 0.0001). However, this variant was not associated with PDR progression. Neither *HIF-1α* rs11549465 nor *VEGF* rs3025039 genetic variants were associated with the PDR progression. The circulating AGEP, VCAM-1, HIF-1α, and VEGF were significantly elevated (*p* < 0.0001) while the CTRP3 was significantly decreased (*p* < 0.0001) in NPDR and PDR groups. The *HIF-1α* rs11549465 CT and/or TT genotype carriers were significantly associated with AGEP and VCAM-1 levels in the NPDR group, while it showed a significant association with the CTRP3 level in the PDR group. The *VEGF* rs3025039 TT genotype carriers showed only a significant association with the CTRP3 level in the PDR group.

**Conclusion:**

The significant association of *HIF-1α* rs11549465 other than *VEGF* rs3025039 with the initiation of NPDR in T2DM Egyptian patients might protect them from progression to the proliferative stage *via* elevating circulating HIF-1α. However, this protective role was not enough to prevent the development of NPDR because of enhancing angiogenesis and inflammation together with suppressing anti-inflammation. The non-significant association of *HIF-1α* rs11549465 with PDR among T2DM patients could not make this variant a risk factor for PDR progression.

## Background

Diabetic retinopathy (DR) is one of the important microvascular complications of T2DM affecting over 30% of diabetic patients. DR is one of the leading causes of blindness among adults worldwide affecting patients’ quality of life and having negative social and psychological effects [1]. In 2015, about 42% of Egyptian diabetic patients have DR and 5% of them become blind [2]. The number of diabetic patients in Egypt is rising and is expected to shift up to eighth in 2030 and seventh in 2045 which means increasing the number of patients with retinopathy as a diabetic complication [3].

Diabetic retinopathy has two stages: early-stage non-proliferative diabetic retinopathy (NPDR) and a more advanced and aggressive proliferative diabetic retinopathy (PDR). NPDR is characterized by high vascular permeability and capillary occlusion in association with the presence of microaneurysms, hemorrhages, and hard lipid exudates in the fundus examination. PDR is marked by the growth of new and abnormal vessels into the vitreous scaffold leading to vitreous hemorrhage and consequently sudden loss of vision. This neovascularization can lead to the fibrosis of the inner retinal surface and contract causing tractional retinal detachment [4, 5].

Genetic factors are implicated in the development of DR making them the most important in DR pathogenesis. These factors are considered significant risk factors in 25–50% of the development and progression of DR. Therefore, genetic relationship studies are useful for the identification of the genetic variants impacting the pathogenesis of DR [5, 6]. Single nucleotide polymorphisms (SNPs) account for 90% of DNA polymorphisms; most of them are functionally neutral that can regulate the gene expression or the function of the coded protein, which therefore contribute to the severity of diseases among individuals [7]. Numerous SNPs have been suggested to be implicated in the pathogenesis of DR including hypoxia-inducible factor-1 (*HIF-1α*) and vascular endothelial growth factor (*VEGF*) genes, some of which have been reported to control their proteins expression [8, 9].

Retinal hypoxia has a key role in the pathogenesis of DR via causing inflammation, oxidative stress, vascular dysfunction, pericyte loss, and pathological neovascularization [10]. HIF-1 is the key mediator in cellular oxygen homeostasis that facilitates the adaptation to retinal hypoxia by regulating the expression of genes that are involved in cellular energy metabolism and glucose transport, angiogenesis, and erythropoiesis [11]. The alpha subunit of HIF-1 is oxygen-sensitive and functions as a master regulator of various genes involved in the hypoxic pathway [9]. The human *HIF-1α* gene locates at chromosome 14q23.2 and composes of 15 exons. Many SNPs could control the differential expression of *HIF-1α*. Among them, the missense SNP (1772 C/T allele of rs11549465) within the oxygen-dependent degradation domain has been reported to regulate the *HIF-1α* expression [12].

The VEGF is responsible for the initial changes in DR involving the breakdown of the blood-retinal barrier, macular edema, and eye neovascularization [13]. The *VEGF* gene is located on chromosome 6p21.3 and is composed of eight exons separated by seven introns [14]. Many SNPs could control the differential expression of *VEGF* [8, 15]. Among them, the +936C/T allele of rs3025039 in the gene promoter region is the main loci [16] that can regulate the *VEGF* expression [17].

Several biomarkers have been identified to be associated with endothelial dysfunction in DR, such as oxidative stress through the production of advanced glycosylation end products (AGEP), chronic inflammation [18, 19], and dysregulated growth factors and cytokines [19, 20]. Vascular cell adhesion protein-1 (VCAM-1) cytokine plays a key role in the inflammatory process via inducing vascular hyperpermeability and blood-retinal barrier breakdown [21]. Oxidative stress can directly or indirectly stimulate the release of proinflammatory cytokines and VEGF resulting in retinal vascular damage, pathological neovascularization, and consequently the development of DR [22, 23].

The C1q complement/TNF-related proteins (CTRPs) family are adipokines that have metabolic effects similar to adiponectin, particularly CTRP3, and may serve as prognostic markers for diseases [24]. CTRP3 is an anti-inflammatory adipokine [24, 25] that promotes cellular differentiation and proliferation, increases hepatic lipid oxidation and adipokine secretion, and attenuates inflammation response [26]. This adipokine serves as a novel diagnostic biomarker and independent predictor for DR [27]. A recent study by Yan et al. [28] reported the therapeutic potential of CTRP3 on the diabetic retina by reducing the vascular permeability, preserving the function of the inner blood-retinal barrier in NPDR via inhibiting diabetes-suppressed the expression of Occludin and Claudin-5 in an AMPK-dependent manner.

To the best of our knowledge, the current study was undertaken for the first time to investigate the predictive roles of *HIF-1α* rs11549465 and *VEGF* rs3025039 SNPs as risk factors for the development of DR among T2DM patients. In addition, the association of these SNPs with the levels of AGEP, VCAM-1, HIF-1α, VEGF, and CTRP3 in serum was investigated to understand their effects on the progression of retinopathy among diabetic patients.

## Methods

### Sample size calculation

The sample size was calculated using the *χ*^2^ test. It was assumed that there are no associations between the studied variables and diabetic retinopathy in Egyptians as a null hypothesis. While associations between the studied variables and diabetic retinopathy in Egyptians were assumed as the alternative hypothesis. The power of the study was supposed to be 80%, α error (two-sided) of 0.05, β error of 0.2, and the effect size (odds ratio of 3).

It was postulated that P1 = proportion of patients expected to have the studied variables in the diabetic retinopathy while P2 = proportion of control subjects having the studied variables (P2) and the authors found it to be about 0.1. The value of P1 was estimated from the equation of P1 = (OR × P2)/[(1 − P2) + (OR × P2)] and found to equal 0.3. Based on this estimation, a total of 72 patients were enrolled per group in the current study.

### Patients

The current retrospective study was approved by the ethical committee of the Research Institute of Ophthalmology (RIO), Giza, Egypt. All procedures were conducted under the Declaration of Helsinki. Written informed consent was obtained from all subjects before participating in this study. Seventy-two control subjects were recruited from the refractive clinic of RIO during their annual refractive check-up. A total of 216 patients with T2DM were enrolled from October 2019 to March 2021 at the medical retina outpatient clinic of the RIO institute. These patients were diagnosed based on fasting plasma glucose (FPG) levels and glycated hemoglobin (HbA1c) percentages according to the criteria of the World Health Organization (WHO/IDF 2006). Among the diabetic patients, 72 patients were without retinopathy (DWR), other 72 diabetic patients were suffered from non-proliferative diabetic retinopathy (NPDR), and the other 72 patients were progressed to proliferative diabetic retinopathy (PDR).

The inclusion criteria were patients with T2DM, aged 18 years or older, and diagnosed at least 5 years before the time of their enrollment in the study. The T2DM patients were chosen to have controlled hypertension. On the other hand, the exclusion criteria were patients with T1DM*,* hepatic diseases, and patients with local eye diseases such as cataracts, glaucoma, or uveitis. In addition, diabetic patients with other complications including diabetic foot, nephropathy, and cardiovascular diseases were excluded.

### Ophthalmic examination

All the patients had a complete ophthalmological examination including a review of medical history, measurement of best-corrected visual acuity using the Snellen chart, slit-lamp examination of the anterior segment, intraocular pressure measurement using Goldmann applanation Tonometer (HAAG-STREIT Bern, Swiss-made), detailed fundus examination (through the dilated pupil) using slit-lamp biomicroscopy and indirect ophthalmoscopy. Fundus fluorescein angiography was done using (Topcon TRC-50DX, Japan). Classification of severity of retinopathy was based according to the Early Treatment Diabetic Retinopathy Study (ETDRS) standardization protocols [29].

### Sample collection

Eight milliliters of venous blood were withdrawn and divided into 3 tubes: Two ml of blood were collected into a sterile ethylene diamine tetraacetic acid (EDTA) vacutainer tube for the assay of glycated hemoglobin as well as *HIF-1α* rs11549465 C> and *VEGF* rs3025039 C>T genotypes, 2 ml blood were collected into a fluoride vacutainer tube for determination of plasma glucose, and the other 4 ml were collected in a plain tube to obtain serum for the remaining laboratory investigations.

### Laboratory investigations

The FPG was assayed using a commercial colorimetric kit (Biomerieux, France) while the HbA1c was analyzed in blood via the ion exchange resin method using a commercial assay kit provided by NS Biotec (Egypt). The levels of triacylglycerol (TG), total cholesterol (TC), low-density lipoprotein cholesterol (LDL-C), high-density lipoprotein cholesterol (HDL-C), and very-low-density lipoprotein cholesterol (VLDL-C) were assayed in serum using commercial assay kits (Spectrum, Egypt).

### Assays of HIF-1α (rs11549465) and VEGF rs3025039 SNPs

The *HIF-1α* rs11549465 C>T and *VEGF* rs3025039 C>T SNPs were genotyped using polymerase chain reaction-restriction fragment length polymorphism (PCR–RFLP) technique. Briefly, genomic DNA was extracted from the whole blood using the genomic DNA extraction kit (Thermo Fisher Scientific, USA) according to the manufacturer’s instructions. The concentration and the purity of the isolated DNA were assessed by measuring the optical density at 260 and 280 nm using NanoDrop™ 2000 spectrophotometer (Thermo Fisher Scientific, USA).

A total amount of 100 ng of the purified genomic DNA was amplified in a volume of 25 μl reaction mixture containing 12.5 μl of 2× PCR master mix (Thermo Fisher Scientific, USA), 1 μl of each of the forward and the reverse primers (Thermo Fisher Scientific, USA) for the gene of interest, and then the reaction’s volume was completed with nuclease-free water. PCR tubes were loaded into Biometra™ Thermal Cycler, USA. The PCR conditions were as follows: 10 min at 94 °C for initial denaturation followed by 40 amplification cycles of denaturation at 94 °C for 30 s, annealing at 59 °C for *HIF-1α* or 64 °C for *VEGF* for 1 min, and extension at 72 °C for 10 min. The sequences of the primers used for *HIF-1α* rs11549465 C>T used were 3′-GGGTAGGAGATGGAGATGCAATCA-5′ forward primer and 5′-GCTGAAGACACAGAAGCAAAGAAC-3 reverse primer [30]. Moreover, forward primer sequence of 3′-AAGGAAGAGGAGACTCTGCGCAGAGC-5′ and reverse primer sequence of 5′-TAAATGTATGTATGTGGGTGGGTGTGTCTACAGG-3′ were utilized for *VEGF* rs3025039 C>T [31].

The PCR products were analyzed by electrophoresis on a 2% agarose gel (Gel Electrophoresis unit Biometra Compact M, Germany). The size of the PCR amplified products was 467 bp for *HIF-1α* rs11549465 C>T and 208 bp for *VEGF* rs3025039 C>T. Ten microliters of the amplified PCR products was then subjected to restriction digestion at 37 °C for 2 h using 1 μl of *HphI* (10U/μl) for *HIF-1α* rs11549465 C>T and *NlaIII* (5 U/μl) for *VEGF* rs3025039 C>T. *HphI* and *NlaIII* were purchased from Thermo Fisher Scientific (USA). The digested products were visualized on 4% agarose gel stained with ethidium bromide.

**Fig. 1:**
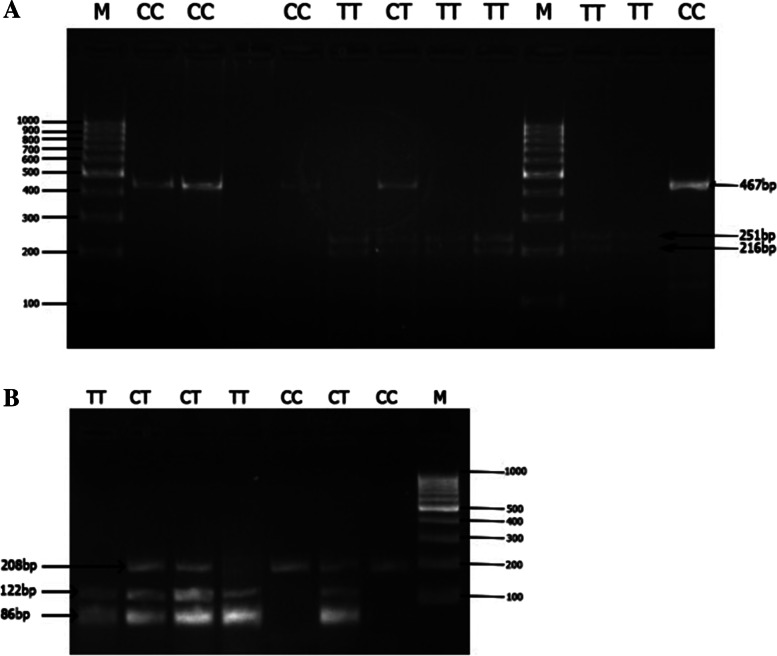
Agarose gel electrophoresis showing RFLP analysis. **A** *HIF-1α* rs11549465 C>T. **B** *VEGF* rs3025039 C>T. M: Molecular weight marker

On the digestion with *HphI*, the amplified product of *HIF-1α* rs11549465 C>T produced an undigested product length of 467 bp for the wild-type CC genotype, 467-, 251-, and 216 bp fragments for CT genotype (heterozygous), and 251- and 216-bp fragments for TT genotype (homozygous mutant) (Fig. 1A).

Regarding the digestion with *NlaIII*, the amplified product of *VEGF* rs3025039 C>T yielded an amplified product of the length of 208 bp for the wild-type CC genotype, 208-, 122-, and 86-bp fragments for CT genotype, and 122- and 86-bp fragments for TT genotype (Fig. 1B).

### Assay of angiogenic, inflammatory, and anti-inflammatory markers

Serum levels of AGEP, VCAM-1, HIF-1*α*, VEGF, and CTRP3 were estimated using enzyme-linked immunoassay research kits purchased from Cell Biolabs’ OxiSelect™, USA.

### Statistical analysis

All statistical analyses were performed using the Statistical Package for Social Science version 26 for Windows (SPSS software package, Chicago, USA). The distribution of data was statistically determined using the Kolmogorov-Smirnov test with Lilliefors significance correction. The categorical variables were expressed as frequencies (percentages), parametric data were expressed as mean ± standard deviation, and non-parametric data were expressed as median (interquartile range). Parametric variables were analyzed between the different studied groups using a one-way ANOVA test followed by Duncan post hoc analysis for multiple comparisons. The non-parametric variables were statistically analyzed using the Kruskal-Wallis one-way ANOVA (K sample) test for multiple comparisons. *χ*^2^ test was used to evaluate both the Hardy–Weinberg equilibrium (HWE) and the differences in allele frequencies for each SNP between the different studied groups.

Binary logistic regression analyses were used to investigate the strength of the association of *HIF-1α* rs11549465 C>T and *VEGF* rs3025039 C>T variants with the susceptibility to DR between either the NPDR or PDR groups on one side and the DWR group on the other side. The strength of associations was measured by the odds ratio (OR) with a 95% confidence interval (CI) adjusted for FBG, HbA1c, BMI, TG, TC, LDL, HDL, and VLDL. Multiple linear regression analysis was used to assess the association of the independent variables relative to the dependent variables after transforming non-parametric variables into the logistic scale. The regression models were constructed using the “enter” analysis. All *p* values were two-sided, and a *p* value < 0.05 was considered statistically significant. Significance had been adjusted by Bonferroni correction for multiple biomarkers analyses. For association analyses, the statistical significance thresholds were set to *p* < 0.025 after Bonferroni correction.

## Results

The demographics of all individuals in the studied groups are listed in Table 1. The age of control subjects was 35.86 ± 9.25 years. DWR, NPDR, and PDR patients aged 39.74 ± 7.94, 38.177 ± 8.25, and 43.04 ± 9.66, respectively. The frequency of male and female gender was 50% for each in all the studied groups. Multiple comparisons showed significant elevations in BMI in NPDR, and PDR patients when compared to each other and when compared with both control and DWR groups (*P* < 0.0001). In addition, the duration of diabetes was 7, 16, and 12 years in DWR, NPDR, and PDR, respectively. All NPDR patients were under ocular medications while 37 of 72 PDR patients were under ocular medications.

**Table 1:** Demographic characteristics of subjects in the different studied groups

	Control (*n* = 72)	DWR (*n* = 72)	NPDR (*n* = 72)	PDR (*n* = 72)	*F*	*P* value
Age (years)	35.86 ± 9.25^a^	39.74 ± 7.94^b^	38.17 ± 8.25^ab^	43.04 ± 9.66^c^	8.82	< 0.0001
Gender *n* (%)						
Male	36 (50)	36 (50)	36 (50)	36 (50)		
Female	36 (50)	36 (50)	36 (50)	36 (50)		
SBP (mm Hg)	120.00	120.00	120.00	120.00		
DBP (mm Hg)	80.00	80.00	80.00	80.00		
BMI (kg/m^2^)	28.93 ± 5.7^a^	33.03 ± 5.03^b^	37.39 ± 10.43^c^	34.38 ± 5.97^b^	17.52	< 0.0001
Duration of diabetes (years)		7.00 (4.00)	16.00 (4.00)	12.00 (4.00)		
Ocular medications (yes/no)	0/72	0/72	72/0	37/35		

Data are expressed as mean ± SD for parametric variables and frequencies (percentages) for categorical variables

*DWR* diabetes without retinopathy, *PDR* proliferative diabetic retinopathy, *NPDR* non-proliferative retinopathy, *SBG* systolic blood pressure, *DBP* diastolic blood pressure, *BMI* body mass index

Different characters denote significance at *p* < 0.0001

The mean difference is significant at *p* < 0.05

All diabetic patients with and without retinopathy had hyperglycemia manifested by significantly gradual elevations in both FBG level and HbA1c percentage at *P* < 0.0001 (Table 2). In addition, NPDR and PDR patients exhibited hyperlipidemia, compared to the control subjects marked by significant raise (*P* < 0.0001) in the serum levels of TG, TC, LDL-C, and VLDL-C accompanied by a significant reduction (*P* < 0.0001) in the serum HDL-C level.

**Table 2:** Biochemical characteristics of subjects in the different studied groups

	Control (*n* = 72)	DWR (*n* = 72)	NPDR (*n* = 72)	PDR (*n* = 72)
FPG (mg%)	83.00 (18.00)	187.00 (83.50)	283.50 (27.25)	307.00 (50.00)
***P*** **value**		**< 0.0001**^**a**^	**< 0.0001**^**a, b**^	**< 0.0001**^**a, b**^**/0.034**^**c**^
HbA1C (%)	4.80 (0.67)	7.14 (1.18)	7.89 (1.22)	9.74 (2.02)
***P*** **value**		**< 0.0001**^**a**^	**< 0.0001**^**a**^**/0.677**^**b**^	**< 0.0001**^**a, b**^**/< 0.0001**^**c**^
TG (mg%)	100.00 (70.75)	189.00 (40.75)	162.00 (45.25)	178.00 (49.50)
***P*** **value**		**< 0.0001**^**a**^	**< 0.0001**^**a**^**/0.001**^**b**^	**< 0.0001**^**a**^**/0.242**^**b**^**/0.516**^**c**^
TC mg%)	187.00 (79.50)	226.00 (74.00)	220.50 (62.75)	270.00 (90.75)
***P*** **value**		**< 0.0001**^**a**^	**0.105**^**a/**^**0.389**^**b**^	**< 0.0001**^**a**^**/0.151**^**b**^**/< 0.0001**^**c**^
HDL-C (mg%)	93.50 (29.50)	79.50 (37.25)	68.50 (38.75)	65.50 (53.75)
***P*** **value**		**0.468**^**a**^	**< 0.0001**^**a**^**/0.008**^**b**^	**< 0.0001**^**a**^**/0.001**^**b**^**/1.000**^**c**^
LDL-C (mg%)	76.50 (49.90)	115.80 (54.15)	122.80 (78.35)	155.40 (116.60)
***P*** **value**		**0.003**^**a**^	**< 0.0001**^**a**^**/1.000**^**b**^	**< 0.0001**^**a**^**/0.005**^**b**^**/0.024**^**c**^
VLDL-C (mg%)	20.00 (14.15)	37.80 (8.15)	32.40 (9.05)	35.60 (9.90)
***P*** **value**		**< 0.0001**^**a**^	**< 0.0001**^**a**^**/0.001**^**b**^	**< 0.0001**^**a**^**/0.242**^**b**^**/0.516**^**c**^

Data are expressed as median (interquartile range)

*DWR* diabetes without retinopathy, *NPDR* non-proliferative retinopathy, *PDR* proliferative diabetic retinopathy, *FPG* fasting plasma glucose, *HbA1c* glycated hemoglobin, *TG* triacylglycerol, *TC* total cholesterol, *HDL-C* high-density lipoprotein cholesterol, *LDL-C* low-density lipoprotein cholesterol, *VLDL-C* very-low-density lipoprotein cholesterol

^a^Significance versus control group

^b^Significance versus DWR group

^c^Significance versus NPDR group

Significance had been adjusted by Bonferroni correction for multiple tests

The mean difference is significant at *p* < 0.05

In the comparison with the DWR group, the NPDR patients showed an insignificant difference in the serum levels of TC and LDL-C (*P* > 0.05) but they had more significant increases in the TG and VLDL-C levels at *P* = 0.001 in association with a more significant reduction in the HDL-C level (*P* = 0.008). Concerning the PDR group, an insignificant difference was recorded in the serum levels of TG, TC, and VLDL-C (*P* > 0.05) but they exhibited a significant reduction in the HDL-C (*p* = 0.001) and a significant elevation in the LDL-C level (0.005). By comparing the PDR group with the NPDR group, they had an insignificant difference in the TG, TC, HDL-C, and VLDL-C (*p* > 0.05) but they showed a more pronounced significant elevation in the LDL-C level (*p* = 0.024).

The genotypic and allelic frequency of *HIF-1α* and *VEGF* polymorphisms among individuals are summarized in Table 3. The genotypic spread of the two polymorphisms was compatible with HWE in all the studied groups (*p* > 0.05). Compared to the control subjects, DWR patients had no significant difference in the genotype distribution of *HIF-1α* rs11549465 C>T SNP (*p* = 0.079); however, DWR patients showed a significantly higher T allele frequency (*p* = 0.011).

**Table 3:** Genotype distribution and allele frequency of the studied genes in the studied groups

		Control (*n* = 72)	DWR (*n* = 72)	NPDR (*n* = 72)	PDR (*n* = 72)
HIF-1α rs11549465 C>T					
Genotype distribution, *n* (%)	CC	47 (65.3)	35 (48.6)	20 (27.8)	25 (34.7)
	CT	21 (29.2)	27 (37.5)	29 (40.3)	29 (40.3)
	TT	4 (5.6)	10 (13.9)	23 (31.9)	18 (25)
	*p-*HWE	0.344	0.141	0.054	0.062
***P*** **value**			**0.079**^**a**^	**< 0.0001**^**a**^**/0.01**^**b**^	**< 0.0001**^**a**^**/0.134**^**b**^**/0.558**^**c**^
Allele frequency (%)	C	80.6	67.4	47.9	54.9
	T	19.4	32.6	52.1	45.1
***P*** **value**			**0.011**^**a**^	**< 0.0001**^**a**^**/< 0.0001**^**b**^	**< 0.0001**^**a**^**/0.03**^**b**^**/0.238**^**c**^
*VEGF* rs3025039 C>T					
Genotype distribution, *n* (%)	CC	21 (29.2)	21 (29.2)	17 (23.6)	21 (29.2)
	CT	30 (41.7)	33 (45.8)	33 (45.8)	29 (40.3)
	TT	21 (29.2)	18 (25)	22 (30.6)	22 (30.6)
	*p-*HWE	0.095	0.410	0.427	0.051
***P*** **value**			**0.830**^**a**^	**0.746**^**a**^**/0.663**^**b**^	**0.980**^**a**^**/0.720**^**b**^**/0.712**^**c**^
Allele frequency (%)	C	52.8	52.1	45.1	49.3
	T	47.2	47.9	54.9	50.7
***P*** **value**			**0.906**^**a**^	**0.195**^**a**^**/0.238**^**b**^	**0.003**^**a**^**/0.637**^**b**^**/0.479**^**c**^

Data are expressed as frequencies (percentage)

*DWR* diabetes without retinopathy, *NPDR* non-proliferative retinopathy, *PDR* proliferative diabetic retinopathy, *HIF-1α* hypoxia-inducible factor-1 alpha, *p-HWE p* value of Hardy–Weinberg equilibrium, *VEGF* vascular endothelial growth factor

^a^Significance versus control group

^b^Significance versus DWR group

^c^Significance versus NPDR group

*P* < 0.05 was considered significant

On the other hand, NPDR patients showed a significantly higher frequency of CT and TT genotypes with a decrease in CC genotype compared to both the control subjects (*p <* 0.0001) and DWR patients (*p* = 0.01). NPDR patients had a significantly higher T allele frequency (*p <* 0.0001), compared to both control and DWR groups. Additionally, PDR patients showed a significantly higher frequency of CT and TT genotypes with a reduction in CC genotype when compared to the control subjects (*p <* 0.0001). The genotype distribution in PDR group showed no statistical significance compared to both DWR (*p* = 0.134) and NPDR (*p* = 0.558) groups. Regarding T allele frequency in the PDR group, it was significantly higher, compared to both the control individuals (*p* < 0.0001) and DWR patients (*p* = 0.03) with insignificant T allele frequency, compared to the NPDR group (*p* > 0.05).

Concerning *VEGF* rs3025039 C>T SNP, the results showed no significant difference in the genotype distribution and allele frequency either in the control, DWR, or NPDR groups. PDR patients had no significant difference (*p* > 0.05) in the genotype distribution when compared to the control, DWR, and NPDR groups. However, PDR patients showed a significantly higher T allele frequency (*p* = 0.003), compared to the control group, without statistical differences (*p* > 0.05), compared to DWR and NPDR groups.

Results in Table 4 showed a significant association of *HIF-1α* rs11549465 C>T (recessive model) with the risk of NPDR development (*p* = 0.022) among T2DM, compared with the DWR group. However, The SNPs of *HIF-1α* rs11549465 C>T at the homozygous, heterozygous, and dominant models as well as *VEGF* rs3025039 C>T at all genetic models were not associated with the development of NPDR (*p* > 0.025). On the other hand, no significant associations of either *HIF-1α* rs11549465 C>T or *VEGF* rs3025039 C>T SNPs were recorded with the risk of PDR progression under any of the tested genetic models, compared with the DWR group. The same results were observed when comparing the PDR group with the NPDR group.

**Table 4:** Association of *HIF-1α* and *VEGF* variants with diabetic retinopathy risk according to genetic association models using binary logistic regression

	NPDR vs. DWR		PDR vs. DWR	
	^#^Adjusted OR (95% CI)	*P* value	^#^Adjusted OR (95% CI)	*P* value
*HIF-1α* rs11549465 C>T				
Homozygous model (TT versus CC)	0.00 (0.00–0.00)	0.968	0.00 (0.00–0.00)	0.999
Heterozygous model (CT versus CC)	0.23 (0.01–4.10)	0.314	14.42 (0.06–3689)	0.346
Dominant model (CC/CT versus CC)	2.15 (0.37–12.49)	0.396	7.87 (0.03–1973)	0.464
Recessive model (TT versus CT/CC)	42.82 (1.72–1007)	0.022	0.51 (0.01–43.92)	0.764
*VEGF* rs3025039 C>T				
Homozygous model (TT versus CC)	0.00 (0.00–0.00)	0.972	0.00 (0.00–0.00)	0.978
Heterozygous model (CT versus CC)	10.88 (0.74–159.86)	0.082	0.01 (65.73)	0.813
Dominant model (CC/CT versus CC)	18.72 (1.42–246.14)	0.026	0.45 (0.003–61.72	0.749
Recessive model (TT versus CT/CC)	7.77 (0.65–93.22)	0.106	3.02 (0.001–6181)	0.776

*DWR* diabetic without retinopathy, *NPDR* non-proliferative retinopathy, *PDR* proliferative retinopathy, *OR* odds ratio, *95% CI* 95% confidence interval

^#^Adjusted for age, BMI, FBG, HbA1c, TG, TC, LDL, HDL, and VLDL

*P* < 0.025 was considered significant after the Bonferroni correction

Table 5 shows levels of AGEP, VCAM-1, HIF-1α, VEGF, and CTRP3 in serum of the different studied groups. Diabetic patients without retinopathy had significant elevations in the serum levels of AGEP (1077.60%, *p* < 0.0001) and VCAM-1 (223.29%, *p* < 0.0001) in association with a significant reduction in the serum level of CTRP3 (40.09%, *p* < 0.0001), compared to the control subjects. While the serum levels of both HIF-1α and VEGF showed no significant differences (*p* > 0.05), compared to the control group. Compared to the DWR group, both NPDR and PDR patients showed significant increases (*p* < 0.0001) in the serum levels of AGEP, VCAM-1, HIF-1α, VEGF, and a more pronounced significant reduction in the serum level of CTRP3 (*p* < 0.0001). By comparing the PDR group with the NPDR group, no significant changes were recorded in the serum levels of AGEP, VCAM-1, HIF-1α, and CTRP3. In addition, a more pronounced increase in the serum level of VEGF (10.02%, *p* = 0.041) was recorded in the PDR group.

**Table 5:** Levels of serum AGEP, VCAM-1, HIF-1α, VEGF, and CTRP3 in all the studied groups

	Control (*n* = 72)	DWR (*n* = 72)	NPDR (*n* = 72)	PDR (*n* = 72)
AGEP (μg/ml)	19.02 (21.26)	223.96 (155.22)	799.16 (794.37)	786.00 (713.09)
***P*** **value**		**< 0.0001**^**a**^	**< 0.0001**^**a, b**^	**< 0.0001**^**a, b**^**/ 1.000**^**c**^
VCAM-1 (ng/ml)	117.00 (47.65)	378.25 (47.22)	589.90 (399.70)	682.80 (461.80)
***P*** **value**		**< 0.0001**^**a**^	**< 0.0001**^**a, b**^	**< 0.0001**^**a, b**^**/ 0.983**^**c**^
HIF-1α (pg/ml)	8.42 (4.12)	10.28 (4.48)	16.92 (5.19)	18.03 (3.93)
***P*** **value**		**< 0.076**^**a**^	**< 0.0001**^**a, b**^	**< 0.0001**^**a, b**^**/ 1.000**^**c**^
VEGF (pg/ml)	84.43 (4.76)	85.95 (3.25)	89.35 (12.69)	98.30 (6.80)
***P*** **value**		**0.306**^**a**^	**< 0.0001**^**a, b**^	**< 0.0001**^**a, b**^**/ 0.041**^**c**^
CTRP3 (ng/ml)	20.27 (7.97)	10.32 (5.70)	3.16 (2.64)	3.34 (3.53)
***P*** **value**		**< 0.0001**^**a**^	**< 0.0001**^**a, b**^	**< 0.0001**^**a, b**^**/ 1.000**^**c**^

Data are expressed median (interquartile range)

*DWR* diabetes without retinopathy, *NPDR* non-proliferative retinopathy, *PDR* proliferative diabetic retinopathy, *AGEP* advanced glycation end products, *VCAM-1* vascular cell adhesion molecule-1, *HIF-1α* hypoxia-inducible factor-1 alpha, *VEGF* vascular endothelial growth factor, *CTRP3* C1q tumor necrosis factor-related protein 3

^a^Significance versus control group

^b^Significance versus DWR group

^c^Significance versus NPDR group

Significance had been adjusted by Bonferroni correction for multiple tests

The mean difference is significant at *p* < 0.05

**Fig. 2:**
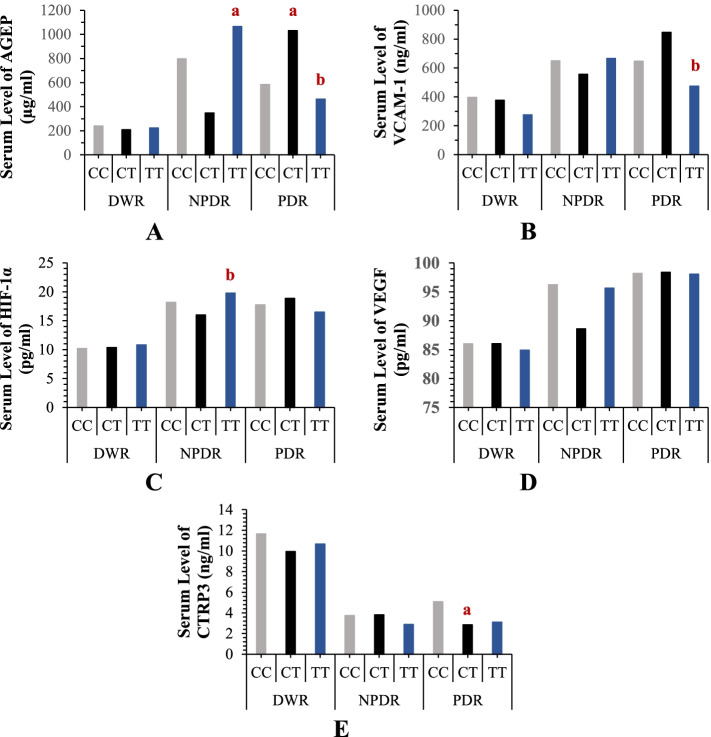
The relationship of the serum levels of **A** AGEP, **B** VCAM, **C** HIF-1α, **D** VEGF, and **E** CTRP3 with the genotypes of *HIF-1α* rs11549465 in diabetic patients. Results are expressed as a median. a: significant compared to CC genotype while b: significant compared to CT genotype. *P < 0.05 was considered significant*

In the NPDR group, TT genotype carriers of *HIF-1α* rs11549465 SNP were significantly associated with the elevation in the serum level of AGEP (*p* = 0.001 when compared to CC genotype), Fig. 2. In addition, the TT genotype carriers were found to be significantly associated with the elevation in the serum level of HIF-1α (*p* = 0.012 when compared to the CT genotype). In the PDR group, CT genotype carriers were significantly associated with the elevation in AGEP (*p* = 0.003 when compared to CC genotype), while TT carriers were significantly associated with the elevations in AGEP (*p* = 0.011) and VCAM-1 (*p* =0.006) when compared to CT carriers. CT genotype carriers showed significant associations with the reduction in the serum level of CTRP3 (*p* = 0.022) when compared to the CT genotype.

**Fig. 3:**
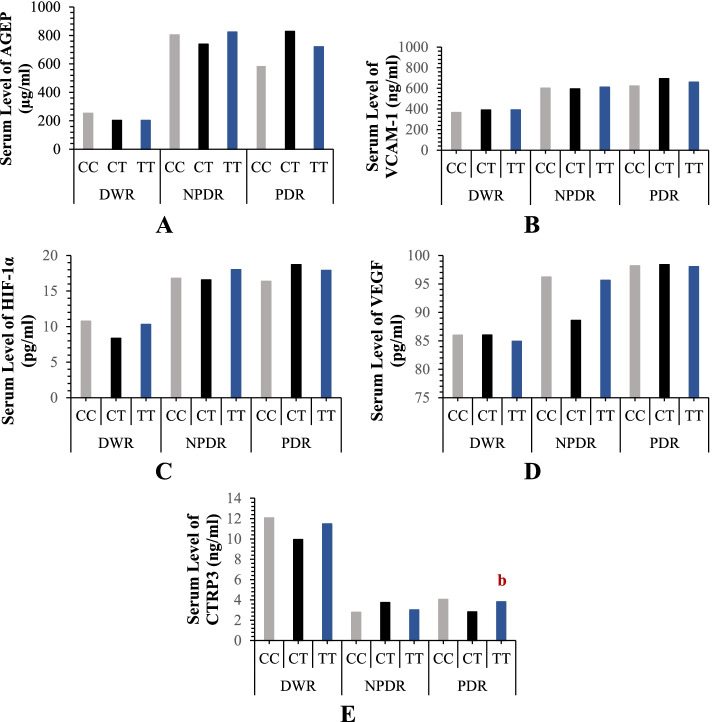
The relationship of the serum levels of **A** AGEP, **B** VCAM, **C** HIF-1α, **D** VEGF, and **E** CTRP3 with the genotypes of *VEGF* rs3025039 in diabetic patients. Results are expressed as a median. a: significant compared to CC genotype while b: significant compared to CT genotype. *P* < 0.05 was considered significant

Data presented in Fig. 3 illustrate that the NPDR group showed no significant associations of all genotypes’ carriers of *VEGF* rs3025039 SNP with the elevations in the serum levels of AGEP, VCAM-1, HIF-1α, and VEGF, as well as the reduction in the CTRP3 level (*p* > 0.05). Concerning the PDR group, the TT genotype carriers were significantly associated with the reduction in the CTRP3 level when compared with the CT genotype (*p* = 0.04).

Table 6 describes the associations of the studied SNPs with their coded protein levels as well as the importance of AGEP, VCAM-1, HIF-1α, VEGF, and CTRP3 as independent variables for FBG and HbA1c as dependent variables among all individuals. There was a significant association of *HIF-1α* rs11549465 SNP with the elevating level of HIF-1α protein (*p* < 0.0001). On the other hand, no significant correlation between *VEGF* rs3025039 SNP and VEGF level was recorded (*p* > 0.05). Serum levels of AGEP, VCAM-1, HIF-1α, and CTRP3 were significantly associated with the elevation in FPG (*p* < 0.0001). While AGEP, VCAM-1, VEGF, and CTRP3 variables showed significant associations with the elevation in HbA1c at *p* < 0.0001, 0.018, and 0.01, respectively.

**Table 6:** Multiple linear regression analysis for the association of SNPs with their protein levels as well as serum levels of biomarkers with both FBG and HbA1c

Dependent variable	Predictors	*R*	*r*^2^	*F*	*P* value^*^	β	*t*	*P* value	VIF
HIF-1α	*HIF-1α* rs11549465	0.24	0.06	17.11	< 0.0001	0.24	4.14	< 0.0001	1
VEGF	*VEGF* rs3025039	0.06	0.003	0.95	0.330	− 0.06	− 0.98	0.330	1
FPG	AGEP (μg/ml)	0.89	0.80	223.80	< 0.0001	0.34	5.98	< 0.0001	4.63
	VCAM-1 (ng/ml)					0.33	5.98	< 0.0001	3.98
	HIF-1α (pg/ml)					0.159	3.99	< 0.0001	2.22
	VEGF (pg/ml)					− 0.05	− 1.53	0.128	1.76
	CTRP3 (ng/ml)					− 0.19	− 4.21	< 0.0001	2.84
HbA1c	AGEP (μg/ml)	0.84	0.71	134.71	< 0.0001	0.46	6.66	< 0.0001	4.63
	VCAM-1 (ng/ml)					0.32	4.89	< 0.0001	3.98
	HIF-1α (pg/ml)					0.05	0.96	0.336	2.22
	VEGF (pg/ml)					− 0.10	− 2.37	0.018	1.76
	CTRP3 (ng/ml)					− 0.14	− 2.59	0.010	2.84

*FPG* fasting plasma glucose, *HbA1c* glycated hemoglobin A1c, *β* standardized coefficient, *VIF* variance inflation factor, *AGEP* advanced glycation end products, *VCAM-1* vascular cell adhesion molecule-1, *HIF-1α* hypoxia-inducible factor-1 alpha, *VEGF* vascular endothelial growth factor, *CTRP3* C1q tumor necrosis factor-related protein 3

^*^P value obtained from ANOVA table

*P* < 0.05 was considered significant

Data shown in Table 7 illustrate the association of AGEP, VCAM-1, HIF-1α, VEGF, and CTRP3 as independent variables for TG, TC, HDL-C, LDL-C, and VLDL-C as dependent variables among all individuals. serum level of AGEP was significantly associated with the elevations in TG (*p* < 0.0001), cholesterol (*p* = 0.031), LDL-C (*p* = 0.021), and VLDL-C (*p* < 0.0001). The serum level of CTRP3 showed a significant correlation with both TG and VLDL-C at *p* = 0.046. There was a significant association between HIF-1α serum level and the elevation of both total cholesterol (*p* = 0.03) and LDL-C (*p* = 0.018). Moreover, the serum level of VCAM-1 had a significant relationship only with the elevation of LDL-C (*p* = 0.005). No associations of the independent variables were recorded with the reduction in the serum level of HDL-C (*p* > 0.05).

**Table 7:** Multiple linear regression analysis for the association of serum levels of AGEP, VCAM-1, HIF-1α, VEGF, and CTRP3 with the lipid profile

Dependent variable	Predictors	*R*	*r*^2^	*F*	*P* value^*^	β	*t*	*P* value	VIF
TG	AGEP (μg/ml)	0.47	0.22	16.31	< 0.0001	0.59	5.21	< 0.0001	4.63
	VCAM-1 (ng/ml)					0.14	1.29	0.198	3.98
	HIF-1α (pg/ml)					− 0.08	− 1.05	0.296	2.22
	VEGF (pg/ml)					− 0.12	− 1.66	0.098	1.76
	CTRP3 (ng/ml)					0.18	2.00	0.046	2.84
Cholesterol	AGEP (μg/ml)	0.31	0.10	6.09	< 0.0001	0.26	2.17	0.031	4.63
	VCAM-1 (ng/ml)					0.22	1.91	0.057	3.98
	HIF-1α (pg/ml)					− 0.18	− 2.19	0.030	2.22
	VEGF (pg/ml)					0.06	0.85	0.395	1.76
	CTRP3 (ng/ml)					0.14	1.45	0.149	2.84
HDL-C	AGEP (μg/ml)	0.30	0.09	5.73	< 0.0001	− 0.07	− 0.60	0.551	4.63
	VCAM-1 (ng/ml)					− 0.11	− 0.93	0.353	3.98
	HIF-1α (pg/ml)					− 0.04	− 0.47	0.638	2.22
	VEGF (pg/ml)					0.09	1.15	0.252	1.76
	CTRP3 (ng/ml)					0.17	1.76	0.079	2.84
LDL-C	AGEP (μg/ml)	0.38	0.15	9.58	< 0.0001	0.28	2.32	0.021	4.63
	VCAM-1 (ng/ml)					0.31	2.86	0.005	3.98
	HIF-1α (pg/ml)					− 0.19	− 2.37	0.018	2.22
	VEGF (pg/ml)					− 0.07	− 0.89	0.373	1.76
	CTRP3 (ng/ml)					0.07	0.72	0.471	2.84
VLDL-C	AGEP (μg/ml)	0.477	0.22	16.32	< 0.0001	0.59	5.21	< 0.0001	4.63
	VCAM-1 (ng/ml)					0.14	1.29	0.198	3.98
	HIF-1α (pg/ml)					− 0.08	− 1.05	0.296	2.22
	VEGF (pg/ml)					− 0.12	− 1.66	0.098	1.76
	CTRP3 (ng/ml)					0.18	2.00	0.046	2.84

*TG* triacylglycerol, *HDL-C* high-density lipoprotein cholesterol, *LDL-C* low-density lipoprotein cholesterol, *VLDL-C* very low-density lipoprotein cholesterol, *β* standardized coefficient, *VIF* variance inflation factor, *AGEP* advanced glycation end products, *VCAM-1* vascular cell adhesion molecule-1, HIF-*1α* hypoxia-inducible factor-1 alpha, *VEGF* vascular endothelial growth factor, *CTRP3* C1q tumor necrosis factor-related protein 3

^*^P value obtained from ANOVA table

*P* < 0.05 was considered significant

## Discussion

The *HIF-1α* rs11549465 SNP (C T resulting in replacement of proline with serine amino acid), has functional importance making it an attractive point for intensive study of its association with various diseases [9]. Furthermore, genetic variants of *VEGF* with dysregulated protein expression are implicated in DR, with markedly raised protein levels [32].

In the current study, the significantly high-frequency distribution of *HIF-1α* rs11549465 TT genotype among NPDR patients accompanied by a significant association at the recessive model with the development of NPDR was observed. This may protect the NPDR patients from progression to the proliferative phase. This association showed crosslinks with the angiogenic, inflammatory, and anti-inflammatory markers.

Different studies illustrated the association of *HIF-1α* rs11549465 variant with DR among T2DM patients. Yamada et al. [33] found that *HIF-1α* rs11549465 SNP was associated with T2DM in Japanese patients but was not associated with the progression of T2DM to retinopathy in their patients. Ekberg et al. [34] reported the protective effect of *HIF-1α* rs11549465 SNP against the incidence of severe NPDR/PDR in the Sweden population. A recent study reported no significant association between this variant and DR progression in Chinese patients with T2DM [35].

The characteristics of T2DM, hyperglycemia, hyperlipidemia, and obesity, were manifested in the current study. Although these are risk factors of DR, also some diabetic patients with uncontrolled diabetes were reported to not develop retinopathy, while advanced retinopathy can be developed in others with relatively good glycemic index [36]. The HIF-1α in diabetic patients with *HIF-1α* rs11549465 SNP is resistant to hyperglycemia-induced inhibition of HIF-1α activity and therefore this SNP has a protective role against the progression of PDR among diabetic patients [34]. These findings explain the current results in which the association of *HIF-1α* rs11549465 SNP with the NPDR patients could prevent them from progressing to PDR even after a long-lasting T2DM (16 years).

The current study reported the significant association of HIF-1α Pro582Ser with an increased level of HIF-1α in serum of NPDR patients. The presence of serine amino acid in position number 582 of the HIF-1α subunit could act independently to convey inducible responses and confer transcriptional activation [37, 38]. The studies of both Fu et al. [39] and Li et al. [40] revealed that HIF-1α Pro582Ser SNP is a stable variant with elevated transcriptional activity resulting in stimulated HIF-1α protein production and activity which is known to increase adaptability for hyperglycemia-induced hypoxia [41–43]. Even with the preserved sensitivity of HIF-1*α* Pro582Ser to the hyperglycemia-induced HIF-1*α* destabilization, the transactivation activity of this SNP is increased [34].

The current study showed a significant association of HIF-1α rs11549465 SNP with NPDR progression among T2DM patients who have suffered from diabetes for 12 years. Despite this long-lasting period, the patients were not progressed to the proliferative stage. On the other hand, this SNP has not been associated with PDR among T2DM patients with 16 years of diabetic duration. These results are in line with the previous studies which reported the protective role of HIF-1α rs11549465 SNP in which it protects T2DM patients when present from the progression of retinopathy to the proliferative stage. While its absence could not protect T2DM patients having a retinopathy diabetic complication from the development to the PDR stage [33, 34]. The lack of this SNP among T2DM patients makes HIF-1α unstable and degraded faster by the effects of hyperglycemia and hyperlipidemia [44–47]. Recently, Zheng et al. [48] reported that the repression of HIF-1α causes the overproduction of mitochondrial ROS in diabetes; the major cause of diabetic complications.

In the current study, the reported significant associations of PDR patients carrying *HIF-1α* rs11549465 CT and/or TT with the changes in the AGEP, VCAM-1, and CTRP3 levels despite the insignificant association of this SNP with the development of PDR may open a new window to understand the development of PDR among T2DM patients.

Although no significant association was recorded in the current study between the *VEGF* rs3025039 variant and the development of both NPDR and PDR among T2DM patients, these patients have significant elevations in the coded VEGF protein level. Moreover, no associations were recorded between the *VEGF* rs3025039 variant and the elevating levels of AGEP, VCAM-1, HIF-1α, and VEGF in the serum of NPDR and PDR patients. On the other hand, PDR patients carrying this genetic variant have a significant reduction in the serum level of CTRP3.

Conflicting studies illustrate the association of the *VEGF* rs3025039 variant with DR among T2DM patients. Previously, a significant association of *VEGF* +936C/T SNP with DR was reported in Korean [49] and Asian populations [50, 51]. Other studies reported no significant associations of this SNP with NPDR or PDR among T2DM patients [51–54].

The elevations in AGEP, VCAM-1, and VEGF observed in the current study in NPDR and PDR groups in association with the reduction in CTRP3 lead to a more understanding of the mechanism of progression of NPDR and PDR among T2DM patients despite the significant association of *HIF-1α* rs11549465 with NPDR development as well as the insignificant association of *HIF-1α* rs11549465 and *VEGF* rs3025039 with both PDR and NPDR.

The accumulation of AGEP in the retinal blood vessel walls has deleterious effects on the retinal endothelial cells (RECs) [18] by elevating their permeability to induce vascular leakage [20]. AGEP can upregulate the gene expression of their receptor (RAGE) in pericytes and microvascular RECs [55]. Activation of RAGE results in the incidence of oxidative stress, enhancing the production of growth factors, adhesion molecules, and pro-inflammatory cytokines [56, 57] and consequently activating the nuclear factor-kappa B [58] and increasing tumor necrosis factor-α production in RECs [59]. In addition, AGEP upregulates VEGF expression in RECs [60] causing angiogenesis, neovascularization, and increasing microvascular permeability which all are implicated in the pathogenesis of retinopathy [61, 62].

Upon induction by hyperglycemia, RECs, pericytes, retinal pigment epithelial cells, astrocytes, Müller cells, and glial cells increase VEGF production [19, 63–66], which has been recognized as a major angiogenic growth factor responsible for the pathologic retinal neovascularization in DR [67]. The VEGF elevation contributes to the progression of T2DM to NPDR and PDR by promoting vessel endothelial cell proliferation, migration, tube formation, and sprouting [68]. Moreover, VEGF is considered a primary initiator of PDR and a potential mediator of NPDR [69]. Furthermore, VEGF modulates DR-associated inflammatory responses at the early stage of DR [70] by enhancing the expressions of proinflammatory cytokines, chemokines, and adhesion molecules [71–73]. VEGF stimulates the leukocyte adhesion to vessel walls through increasing VCAM-1 expression on RECs [74], which is known to be elevated in the serum of patients with NPDR and PDR [75]. VCAM-1 has an angiogenic effect on endothelial cells via its interaction with its ligand and consequently induces the progression of DR [76, 77]. It is reported that hyperglycemia upregulated VCAM-1 expression on blood vessels in the retina in experimental DR in animals [78].

The observed reductions of CTRP3 levels in NPDR and PDR groups, compared to the DWR group in the present study are in line with the previous report of Elsaid et al. [79] in which dysregulation and reduction of the circulating CTRP3 are involved in the progression of *diabetes mellitus*. The reduction of circulating CTRP3 in the NPDR group despite the significant association of the protective *HIF-1α* rs11549465 SNP with NPDR can be considered another explanation for the initiation of NPDR among T2DM patients. Furthermore, the reduced level of serum CTRP3 in PDR together with the absence of *HIF-1α* rs11549465 SNP association may also clarify the progression of T2DM patients to PDR. CTRP3 exhibits anti-inflammatory via attenuating hyperglycemia and hyperlipidemia-induced elevating VCAM-1 expression in the human retinal microvascular endothelial cells. Furthermore, CTRP3 has an anti-apoptotic effect by hindering the oxidative stress and apoptosis induced in the retinal pigment epithelial cell line [28].

## Conclusions

In conclusion, the *HIF-1α* rs11549465 genetic variant not *VEGF* rs3025039 was reported for the first time to be significantly associated with the development of NPDR in Egyptian patients with T2DM. This association might protect the NPDR patients from progression to the proliferative stage even after long-lasting diabetes because of elevating circulating HIF-1α Pro582Ser which is known to be resistant to hyperglycemia and hyperlipidemia. However, this protective role could not counteract the initiation of NPDR because of the significant elevations in the circulating angiogenic and inflammatory markers as well as the reduction in the anti-inflammatory CTRP3 level. On the other hand, no significant association of *HIF-1α* rs11549465 SNP was reported in the PDR patients which together with the elevations of the angiogenic and inflammatory markers and reduction in the CTRP3 level resulted in the progression of T2DM patients to PDR. Therefore, the *HIF-1α* rs11549465 genetic variant could not be considered as a risk factor for the progression of PDR among T2DM patients.

Future research endorses to study of other *VEGF* genetic variants that could be associated with DR among T2DM in Egyptian patients and correlated with the significant elevation in the circulating VEGF protein level. In addition, further study is recommended to explore the changes in the HIF-1α signaling pathway in T2DM patients carrying *HIF-1α* rs11549465 SNP to better understand the protective role of this SNP against the progression of NPDR to PDR stage.

## Data Availability

The datasets used and/or analyzed during the current study are available from the corresponding author on reasonable request.

## Abbreviations

AGEP: Advanced glycation end product
CI: confidence interval
CTRP3: C1q tumor necrosis factor-related protein 3
DWR: Diabetics without retinopathy
HIF-1α: Hypoxia-inducible factor-1alpha
HWE: Hardy–Weinberg equilibrium
NPDR: Non-proliferative diabetic retinopathy
OR: odds ratio
PDR: Proliferative diabetic retinopathy
PHD: Prolyl hydroxylase domain protein
RECs: Retinal endothelial cells
RFLP: Restriction fragment length polymorphism
SNPs: single nucleotide polymorphisms
VCAM-1: Vascular cell adhesion molecule-1
VEGF: Vascular endothelial growth factor
*VIF*: Variance inflation factor
β: Standardized coefficient
